# Diet Quality and Co-Occurrence of Arteriosclerosis and Knee Osteoarthritis: A Cross-Sectional Survey Among Chinese Iron Workers from Shougang Group

**DOI:** 10.3390/nu18203352

**Published:** 2026-10-13

**Authors:** Ping Yuan, Xin Yuan, Zihao Li, Zhenpeng Guan, Zhiyong Zou, Jing Li

**Affiliations:** 1 Institute of Child and Adolescent Health, School of Public Health, Peking University Health Science Center, Beijing 100191, China; yuanping_632@163.com; 2 National Health Commission Key Laboratory of Reproductive Health, Peking University, Beijing 100191, China; 2010306145@stu.pku.edu.cn; 3 Orthopedic Center, Peking University Shougang Hospital, Beijing 100144, China; lizihao614@163.com (Z.L.); guanzhenpeng@pku.org.cn (Z.G.)

**Keywords:** diet quality, arteriosclerosis, knee osteoarthritis, global dietary recommendations, Chinese, women

## Abstract

Background: Arteriosclerosis (AS) and knee osteoarthritis (KOA) pose a major public health challenge, yet evidence linking diet quality to the risk of comorbidity among Chinese occupational groups remains limited. This cross-sectional occupational study examined the association between diet quality indicators and the co-occurrence of AS and KOA among 1538 iron workers from Shougang Group. Methods: Dietary data were collected using the Diet Quality Questionnaire (DQQ) for China. Multivariable logistic regression models were performed with adjustment for relevant covariates. Results: Overall, 627 workers had co-occurrence of AS and KOA, including 579 men and 48 women. Among women, a higher continuous Global Dietary Recommendations (GDR) score was inversely associated with the odds of co-occurrence (OR = 0.75; 95% CI = 0.62–0.91). Compared with a GDR score ≤ −3, a GDR score ≥ 1 was also inversely associated with co-occurrence among women (OR = 0.13; 95% CI = 0.02–0.77); however, no statistically significant associations were observed among men or in the age- and BMI-stratified analyses. Conclusion: These findings provide preliminary evidence of an inverse association between diet quality and AS–KOA co-occurrence among female workers but should be considered exploratory, requiring confirmation in larger prospective studies.

## 1. Introduction

Multimorbidity, defined as the coexistence of two or more chronic conditions [1], is increasingly observed in working-age populations and has become a widespread public health phenomenon [2]. In China, the prevalence of multimorbidity among older adults currently exceeds 40% [3] and continues to rise, placing a substantial burden on healthcare expenditures [4,5,6].

Given the rising burden of chronic diseases, identifying modifiable risk factors such as diet is crucial. Unhealthy dietary habits are major drivers of mortality from non-communicable diseases (NCDs) worldwide [7]. Healthy dietary patterns, such as the Alternative Healthy Eating Index-2010 (AHEI-2010) [8], Mediterranean diet [9], Dietary Approaches to Stop Hypertension (DASH) [10], and Plant-based Dietary Indices (PDIs) [11], have been demonstrated to reduce chronic diseases risk. A growing body of evidence also links higher dietary quality to a lower number of chronic conditions and slower progression of comorbidity, and a healthy lifestyle has been shown to protect against cardiometabolic comorbidity [2,12,13,14,15,16,17,18].

Previous studies have separately investigated the associations between dietary factors and arteriosclerosis (AS) [19] and between dietary factors and knee osteoarthritis (KOA) [20], whereas evidence regarding diet quality and their co-occurrence remains limited, particularly among industrial workers. Although AS and KOA have distinct and potentially non-synchronous natural histories, both are chronic conditions that develop gradually and may share age-related, metabolic, and inflammatory determinants. Examining their co-occurrence may help identify shared modifiable risk factors and biological pathways that may not be fully understood when each condition is studied in isolation. Such an integrated approach may contribute to a better understanding of complex multimorbidity and inform the coordinated prevention and management of vascular and musculoskeletal conditions. Nevertheless, the presence of both conditions at the same examination indicates contemporaneous co-occurrence and does not imply simultaneous onset, parallel progression, or a shared chronological sequence.

Sex differences exist in nutritional needs, metabolism, and disease susceptibility, influenced by factors including sex hormones and body composition [21]; however, evidence from occupational cohorts of relatively young and middle-aged Chinese adults remains limited. The Shougang Group workforce offers an understudied occupational setting for evaluating diet-related co-occurrence of chronic disease among working-age Chinese adults. Given potential sex differences in nutritional metabolism and disease susceptibility, we pre-specified sex-stratified analyses.

Recently, a dietary quality questionnaire (DQQ) has been developed within a global dietary quality framework to collect dietary data [22]. The DQQ is a standardized instrument defined by its use of dichotomous (yes/no) questions on food and beverage intake to generate diverse dietary quality metrics for population-level assessment [23]. Although preliminary studies exist on diet and chronic diseases, their populations have largely been confined to the elderly, and the DQQ has not been applied in occupational settings. Accordingly, this study was designed to examine the associations between diet quality, as measured by the DQQ, and the co-occurrence of AS and KOA, and its potential variations by sex, among iron workers from the Shougang Group.

To the best of our knowledge, this is the first study in China to investigate the relationship between diet quality and the co-occurrence of AS and KOA in an industrial worker population using the DQQ. Because AS and KOA may share inflammatory, metabolic, and vascular pathways, their co-occurrence provides a clinically relevant endpoint for occupational health research [24,25,26,27,28,29,30,31,32,33,34,35,36,37,38].

Participants were recruited from the routine health-examination population of the Shougang Group, a large industrial enterprise in Beijing. This workforce was selected because of its stable population, standardized health-examination procedures, and concurrently collected dietary, vascular, and musculoskeletal data, which enabled consistent assessment of AS and KOA co-occurrence. Moreover, industrial workers may experience distinctive dietary patterns, cardiometabolic risks, and occupational physical demands relevant to both conditions, while working-age occupational populations remain underrepresented in research on diet quality and multimorbidity. Thus, this population provides a relevant setting for investigating the co-occurrence of vascular and musculoskeletal conditions, although it is not necessarily representative of other occupational groups or the general population.

## 2. Materials and Methods

### 2.1. Data Resources and Study Participants

Data for this study were derived from a routine health examination population at the Shougang Group. This workforce constitutes a stable cohort with a decade of accumulated health examination data and routine medical records, making it an excellent population for scientific research. This cohort serves as a resource platform for future large-scale research on chronic disease prevention and control. This study received ethical approval from the Ethics Committee of Peking University Shougang Hospital (approval number: IRBK-2024-012-01).

**Figure 1:**
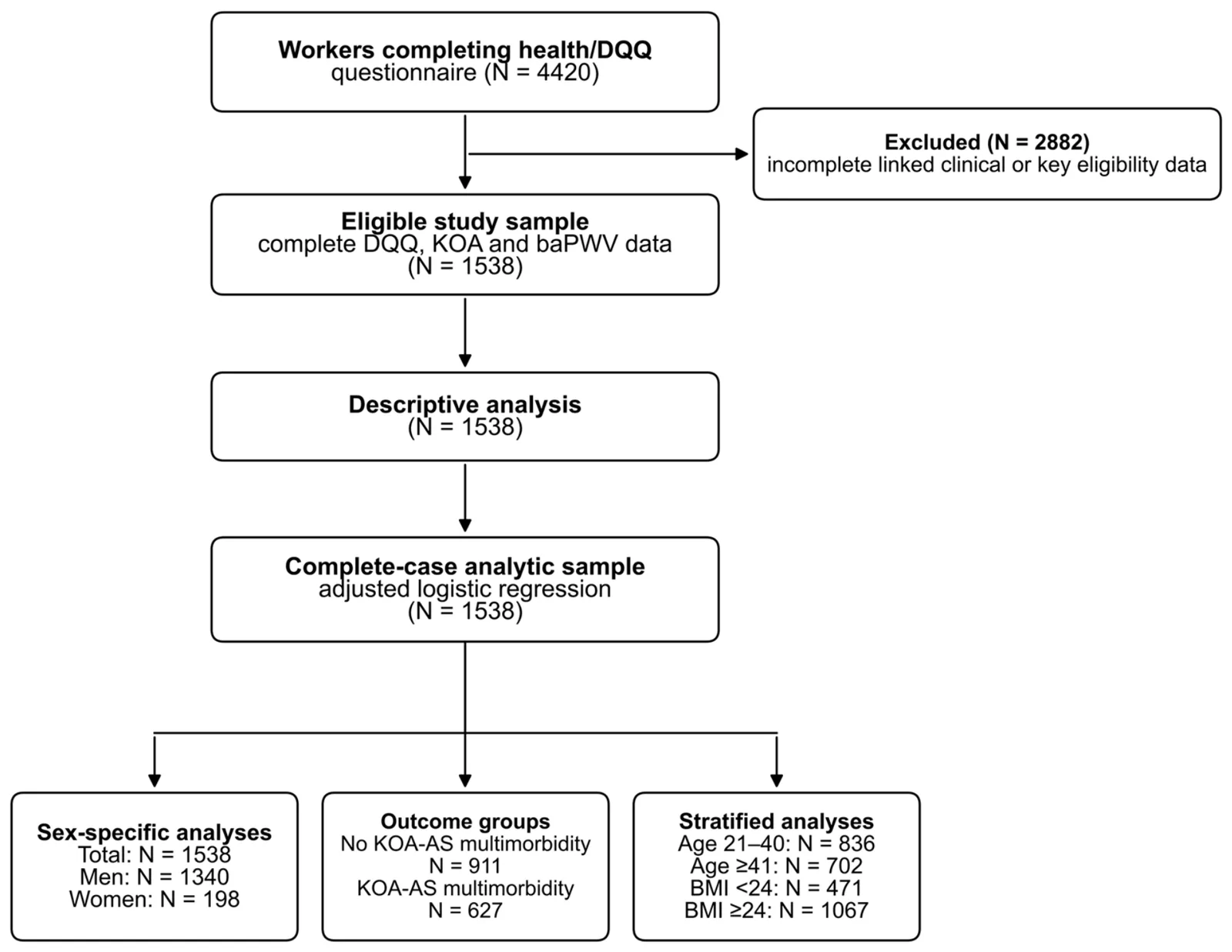
Flowchart of participant selection.

A total of 1538 participants from the 2024 health examination were enrolled (Figure 1). Inclusion criteria were (1) a relatively complete health check-up profile; (2) completion of both knee imaging and vascular brachial-ankle pulse wave velocity (baPWV) examinations; and (3) sufficient literacy to self-complete the survey. The exclusion criteria were (1) ambiguous diagnoses in medical records or missing key study data; (2) presence of severe comorbidities; (3) pregnancy; and (4) any other conditions precluding radiological examinations.

### 2.2. Co-Occurrence and Other Variables

KOA was diagnosed based on relevant guidelines, including the Chinese guidelines for the diagnosis and management of KOA [39], in combination with orthopedic diagnoses from inpatient medical records and imaging findings. The diagnostic criteria were as follows (1) knee pain recurring within the past month; (2) age ≥ 50 years; (3) morning stiffness lasting ≤30 min; (4) crepitus on active joint motion; (5) narrowing of joint space, subchondral sclerosis or cystic changes, or osteophytes at the joint margins shown on X-ray (in standing or weight-bearing positions). A KOA diagnosis required the presence of criterion (1) plus any two of criteria (2)–(5). Cases not meeting these criteria were not classified as KOA.

According to clinical guidelines such as the Chinese Guidelines for Cardiovascular Disease Prevention [40] and the Chinese Guidelines for Hypertension Prevention and Treatment [41], the baPWV measurement can serve as an indicator for assessing AS. AS was diagnosed when the baPWV value was greater than or equal to the age-specific mean reference value plus 2 standard deviations (SD). Individuals with baPWV values greater than or equal the mean plus 1 SD were defined as “suspected AS”. Those not meeting these criteria were considered not to have AS.

The co-occurrence of AS and KOA was defined as simultaneously diagnosed with both KOA and AS according to the methods above. For clarity, KOA and AS were the two components of the prespecified co-occurrence outcome; hypertension and diabetes were not included in the primary outcome definition and were treated as potential confounders. Body mass index (BMI, kg/m^2^) was calculated as weight (kg) divided by height squared (m^2^). Systolic blood pressure (SBP) and diastolic blood pressure (DBP) were measured 2–3 times using a certified upper-arm electronic sphygmomanometer (Omron HEM-7124, Omron Healthcare Co., Ltd., Kyoto, Japan), with one-minute intervals between measurements, and the average value was calculated. Physical activity was assessed using a self-reported questionnaire item and categorized according to the original response options as no exercise, low exercise, occasional exercise, or regular exercise.

### 2.3. Dietary Data Collection and Assessment

Dietary data were collected via self-administration by participants using the Diet Quality Questionnaire (DQQ) tool, designed to assess and monitor the global dietary quality at the population level [22]. The Diet Quality Questionnaire (DQQ) is an internationally validated, low-burden dietary assessment instrument whose food-group-based scoring and categorization thresholds were developed a priori based on 11 global dietary recommendations and validated in nationally representative populations from Brazil and the United States; the present study applied its prespecified categorization to ensure methodological consistency and reproducibility [42]. The China-adapted DQQ utilizes sentinel foods from 29 food groups to effectively represent the dietary patterns of over 95% of the adult population [22].

Four diet quality scores were derived from the DQQ data: the Dietary Diversity Score (DDS), the NCD-Protect score, the NCD-Risk score, and the Global Dietary Recommendations (GDR) score. The DDS refers to the number of food groups consumed the previous day or night of the ten food groups used in the Minimum Dietary Diversity—Women (MDD-W) indicator: (1) grains, white roots and tubers, and plantains, (2) pulses (beans, peas and lentils), (3) nuts and seeds, (4) dairy, (5) meat, poultry, and fish, (6) eggs, (7) dark green leafy vegetables, (8) other vitamin A-rich fruits and vegetables, (9) other vegetables, and (10) other fruits. The NCD-Protect score is an indicator of dietary factors protective against NCDs. It ranges from 0 to 9 points, with 1 point allocated for each predefined food group consumed. The food groups comprised the following nine categories: whole grains, pulses, nuts and seeds, vitamin A-rich orange vegetables, dark green leafy vegetables, other vegetables, vitamin A-rich fruits, citrus fruits, and other fruits. The NCD-Risk score is an indicator of dietary risk factors for NCDs. It ranges from 0 to 9 points, with 1 point allocated for each predefined food group consumed, except that a 2-point value is assigned for processed meat. The food groups comprised the following nine categories: soft drinks, baked/grain-based sweets, other sweets, processed meat, unprocessed red meat, deep fried food, fast food & instant noodles, packaged ultra-processed salty snacks. The GDR score is a metric that indicates adherence to global dietary recommendations, which include dietary factors protective against non-communicable diseases, and it is calculated as the NCD-Protect score minus the NCD-Risk score. Further details on the dietary scores have been reported elsewhere (Supplementary Materials) [43].

Because no established clinical cut-off values are currently available for DQQ-derived diet quality scores in relation to the co-occurrence of AS and KOA, the dietary indicators were analyzed both as continuous variables and as categorical variables. The categorical cut-off points were determined based on the score distributions in the present study population, with reference to previous DQQ studies that applied categorical analyses of diet quality indicators, while also ensuring adequate sample sizes in each category for model stability. Accordingly, the NCD-Protect score was categorized as ≤3, 4–6, and ≥7; the NCD-Risk score as ≤4, 5–8, and 9; the GDR score as ≤−3, −2–0, and ≥1; and the DDS as ≤10, 11–20, and ≥21. These categories should be interpreted as distribution-informed low, intermediate, and high levels rather than clinically validated thresholds.

### 2.4. Statistical Analysis

Data are presented as numbers (percentages) for categorical variables and as medians ± interquartile ranges (IQRs) or means ± SDs for continuous variables (e.g., age, BMI, SBP, and DBP). The Chi-square test, the Wilcoxon rank test, and the *t*-test were used to compare differences in characteristics between men and women. Missing data were handled using complete-case analysis, whereby participants with missing values in any primary study variable (i.e., dietary scores, co-occurrence status, and covariates) were excluded from all statistical analyses. Multivariable logistic regression analyses were used to evaluate the associations of the four diet quality scores (i.e., DDS, NCD-Protect score, NCD-Risk score, and GDR score) with co-occurrence of AS and KOA, and odds ratios (OR) and 95% confidence intervals (CI) were estimated after adjusting for sex, age, ethnicity, BMI, SBP, DBP, smoking, drinking, and physical activity. In sex-, age-, and BMI-stratified analyses, models were re-estimated within each stratum using multivariable adjustment, with the stratification variable omitted when it was constant within a stratum. Because some sex-specific dietary score categories had small cell counts, particularly among women with high GDR scores, Firth penalized logistic regression was additionally performed as a robustness analysis using the same covariate adjustment set. Firth correction was used to reduce small-sample and separation-related bias in maximum likelihood logistic regression estimates. Given the small female subgroup relative to the number of adjustment variables, a prespecified parsimonious female sensitivity model adjusted for age, BMI, and SBP was also fitted using Firth penalized logistic regression. To assess possible over-adjustment from simultaneously including SBP and DBP, additional Firth sensitivity analyses used SBP only, DBP only, or neither blood-pressure variable. To assess potential confounding by lipid-related factors, additional Firth models adjusted for recorded hyperlipidemia and elevated LDL-C (LDL-C ≥ 4.9 mmol/L) were used. Sensitivity analyses were further conducted by excluding participants with hypertension and diabetes simultaneously, and the models were re-estimated using Firth penalized logistic regression; additional sensitivity analyses excluding participants with hypertension only and diabetes only were also performed. Thus, age, BMI, smoking, and physical activity were included as covariates in all primary models. Hypertension and diabetes were not included in the prespecified primary adjustment set because their available disease-history information was not consistently coded across source records; their potential influence was evaluated in sensitivity analyses excluding participants with both conditions and, separately, those with hypertension or diabetes. For comparisons across the four mutually exclusive AS/KOA status groups, separate binary Firth models compared AS, KOA, and AS + KOA with the reference group (participants without either AS or KOA). A two-sided *p*-value < 0.05 was considered statistically significant. Stratification was performed based on age (21–40 years, ≥41 years) and BMI (underweight and healthy weight, overweight and obesity). As this is an exploratory study, no correction for multiple comparisons was applied across the multiple dietary indicators, scoring forms, and subgroup analyses. Nominally significant findings should therefore be interpreted cautiously. Several potential confounders—including socioeconomic status, educational attainment, income, and occupational exposure by job type—were not collected and thus could not be adjusted for. Total energy intake was also not adjusted, as the DQQ is a qualitative tool not designed to quantify energy consumption.

## 3. Results

### 3.1. Participant Characteristics

A total of 1538 participants were included in this study. The cohort was predominantly male (87.13%) and of Han ethnicity (95.58%), with a median age of 40 years. More than one third (34.48%) of the participants reported smoking, and over one tenth (11.38%) reported alcohol consumption, with much higher levels of both smoking and drinking among men compared to women (*p* < 0.001). The mean BMI of the participants was 25.85 ± 3.63 kg/m^2^ (Table S1). Among male workers, participants with AS/KOA co-occurrence were significantly older (median 41 vs. 38 years, *p* = 0.003) and had higher BMI (26.42 vs. 25.59 kg/m^2^, *p* = 0.03), systolic blood pressure (135.85 vs. 125.91 mmHg, *p* < 0.001), diastolic blood pressure (85.30 vs. 79.45 mmHg, *p* < 0.001), and hypertension prevalence (43.52% vs. 17.58%, *p* < 0.001) compared with the reference group. The NCD-Protect score was slightly but significantly lower in the co-occurrence group (median 5 vs. 6, *p* = 0.039), whereas no significant between group differences were observed for the GDR score, DDS, smoking, drinking, or physical activity (Table 1). Among female workers, the co-occurrence group had significantly higher BMI (25.28 vs. 23.97 kg/m^2^, *p* < 0.001), systolic blood pressure (133.06 vs. 120.49 mmHg, *p* < 0.001), diastolic blood pressure (82.15 vs. 74.09 mmHg, *p* < 0.001), and hypertension prevalence (27.08% vs. 8.57%, *p* < 0.001) than the reference group. Notably, the GDR score was significantly lower in women with co-occurrence (median −2 vs. 0, *p* = 0.018), indicating poorer diet quality, whereas no significant differences were found for other dietary indicators, age, smoking, drinking, or physical activity (Table 1).

### 3.2. Distribution of Diet Quality Scores and Co-Occurrence

Table 2 shows that there were 627 cases of co-occurrence of AS and KOA, with 579 in men and 48 in women. Among participants with an NCD-risk score of 9, the percentage of co-occurrence was 44.47% in men and 31.15% in women. Among participants with a GDR score ≤ −3, the percentage of co-occurrence in women was 33.33%. Meanwhile, the subgroup with DDS between 10 and 20 exhibited the highest co-occurrence rate.

### 3.3. Associations of Diet Quality Scores with Co-Occurrence

In the total-sample analyses, the multivariable logistic regression models were adjusted for sex, age, ethnicity, BMI, SBP, DBP, smoking, drinking, and physical activity; sex was omitted from the sex-stratified models. Among women, a higher continuous GDR score was inversely associated with the odds of AS and KOA co-occurrence (OR = 0.75; 95% CI = 0.62–0.91) (Table 3).

Compared with women with a GDR score ≤ −3, those with a GDR score ≥ 1 had lower odds of co-occurrence (OR = 0.13; 95% CI = 0.02–0.77); however, this estimate was based on only three co-occurrence cases and should therefore be considered exploratory and interpreted cautiously (Table 4). Among men, no statistically significant association was observed for a GDR score ≥ 1 compared with a GDR score ≤ −3 (OR = 1.35; 95% CI = 0.94–1.92) (Table 4).

The Firth-corrected robustness analysis yielded results generally consistent with the primary logistic regression models. Among women, the inverse association remained for continuous GDR score (OR = 0.76; 95% CI = 0.63–0.91) and for GDR score ≥ 1 (OR = 0.18; 95% CI = 0.03–0.75) (Supplementary Table S2). As an additional sensitivity analysis addressing the sparse female subgroup, the parsimonious Firth model adjusted for age, BMI, and SBP yielded OR = 0.76 (95% CI = 0.63–0.91) for continuous GDR and OR = 0.17 (95% CI = 0.03–0.74) for GDR ≥ 1 (Supplementary Table S5).

In the sensitivity analysis excluding participants with both hypertension and diabetes, the results were generally consistent with the primary analysis (Table 5). Among women, higher GDR score remained inversely associated with co-occurrence, both as a continuous variable (OR = 0.75; 95% CI = 0.62–0.90) and in the GDR ≥ 1 category (OR = 0.12; 95% CI = 0.01–0.63). Additional analyses excluding participants with hypertension only or diabetes only showed a similar overall pattern (Supplementary Tables S6 and S7). These findings should be interpreted cautiously because of the reduced sample size and small cell counts in sex-stratified analyses (Table 5).

Sensitivity analyses using SBP-only, DBP-only, or neither blood-pressure variable yielded similar overall estimates (Supplementary Tables S8–S10). Across the four mutually exclusive AS/KOA status groups, the results were generally consistent with the primary findings; among women, higher continuous GDR scores were inversely associated with AS + KOA (OR = 0.77; 95% CI = 0.59–0.98) (Supplementary Tables S11–S13). Additional adjustment for recorded hyperlipidemia and elevated LDL-C yielded similar associations for women (Supplementary Table S14).

**Figure 2:**
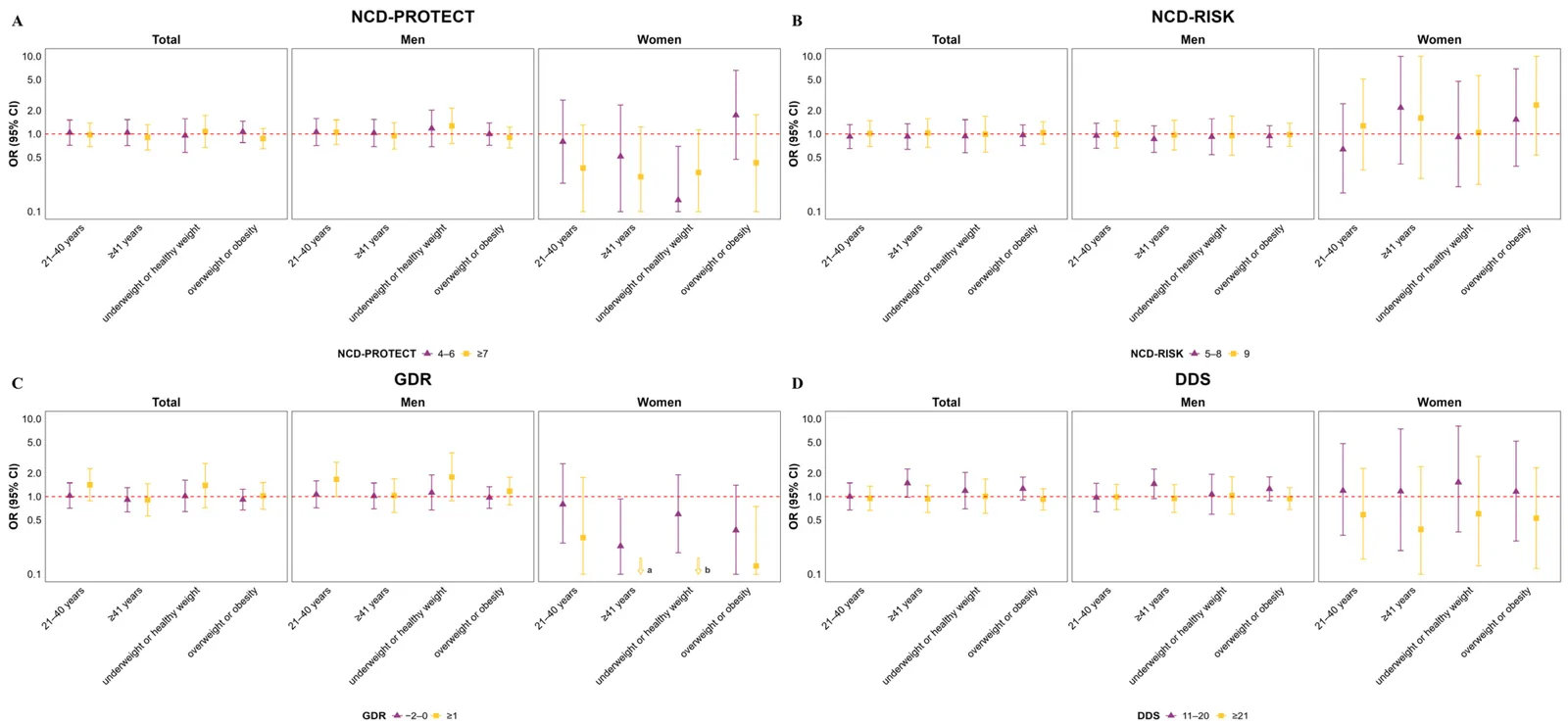
Stratified analysis of the association between diet quality scores and co-occurrence risk by age and BMI. (**A**) NCD-Protect score; (**B**) NCD-Risk score; (**C**) GDR score; (**D**) DDS. Points represent odds ratios (ORs), and error bars indicate 95% confidence intervals (CIs). The red dashed line denotes OR = 1 (no association). Arrows indicate estimates or CI limits outside the displayed range. In (**C**), the 95% CIs were unavailable for women with GDR ≥1 in the ≥41-year (a) and underweight or healthy-weight (b) strata.

Multivariable stratified analyses based on age (21–40 years and ≥41 years) and BMI (<24 kg/m^2^ and ≥24 kg/m^2^) revealed no significant associations between diet quality scores and co-occurrence risk (Figure 2). The models were re-estimated within each stratum and adjusted for the remaining applicable covariates.

Specifically, neither age stratification nor BMI stratification yielded statistically significant relationships across the different subgroups examined. The absence of significant findings in these stratified analyses suggests that the inverse association of higher diet quality observed in the overall female population may not be modified by age or BMI status within this cohort. However, it is important to note that the limited sample sizes within certain strata may have reduced the statistical power to detect potentially meaningful associations, particularly in the female subgroup, where the overall significant association was originally observed.

## 4. Discussion

In this analysis of the 2024 health examination data from 1538 Shougang Group workers, we observed a significant inverse association between the GDR score and the co-occurrence of AS and KOA in female workers, particularly among those with a GDR score ≥ 1. These findings suggest that a higher dietary quality, as captured by the GDR score, is associated with a lower co-occurrence among the female workers. Given that the study population was predominantly male industrial workers from a single steel enterprise, these findings should be interpreted as primarily applicable to male-dominated occupational settings and may not be generalizable to the broader working population.

The biological plausibility of the observed association is supported by evidence that plant-rich dietary patterns may improve vascular function and reduce arterial stiffness through anti-inflammatory, blood pressure lowering and lipid-lowering pathways [44,45,46,47,48], while dietary components such as omega-3 fatty acids, polyphenols, and dietary fiber may have beneficial effects on cartilage homeostasis and KOA-related inflammatory processes [24,25,26,27,28]. However, the significant association observed in this study was confined to the female population. Notably, a recent study using the same DQQ-derived GDR score among 12,051 Chinese adults similarly reported a significant inverse association between higher GDR scores and stroke risk in women (OR = 0.42; 95% CI = 0.22–0.77) but not in men [23], lending cross-disease support to the sex-specific pattern observed here. Research has indicated that following a Mediterranean diet intervention, a significant sex-specific difference in HDL-C levels was observed, with a more pronounced effect of the diet on multiple cardiovascular risk markers evident in women [49]. We speculate that the influence of hormones, such as estrogen, may modulate metabolic and inflammatory responses, mediating a greater sensitivity to dietary components in women [50]. Furthermore, the healthy worker effect has been identified among men but not women, which could lead to an underestimation of the true association between dietary factors and health outcomes in the male workforce. Workload and job assignments may also differ between men and women within the same enterprise; however, detailed job-specific physical demand, standing time, shift work, and lifting information were not available for adjustment. Moreover, the higher rates of smoking and alcohol consumption in men could have obscured the modest association of diet. However, the marked imbalance in sex distribution and the relatively small number of women increased the uncertainty and instability of the sex-specific estimates. In particular, the estimate for women with a GDR score ≥ 1 was based on only three co-occurrence cases and should therefore be considered exploratory. Replication in larger prospective cohorts with adequate representation of women is therefore required.

To our knowledge, this is the first study to examine the association between diet quality and the co-occurrence of AS and KOA among Chinese workers. As an exploratory cross-sectional study, our findings provide preliminary evidence supporting further prospective investigation of this association in occupational populations. Such investigations hold significant promise for informing the development and implementation of targeted dietary interventions in the workplace. However, this study was characterized by an imbalance in sex distribution and a relatively small subsample of women, which primarily increases the uncertainty and instability of effect estimates. Consequently, replication in larger, female-specific cohorts is required.

Nevertheless, several limitations of this study should be acknowledged. First, the study utilizes cross-sectional data from the 2024 health examinations of Shougang Group workers, precluding causal inference; individuals already diagnosed with AS and KOA may have modified their dietary habits before entry; therefore, a higher GDR may reflect disease status rather than causation. In addition, AS and KOA have distinct and potentially non-synchronous natural histories. Because the timing of disease onset and progression was unavailable, the cross-sectional identification of their co-occurrence cannot establish which condition developed first, whether their progression overlapped, or whether diet quality preceded either condition. Longitudinal studies with repeated vascular and joint assessments are needed to characterize the chronological relationships among diet quality, AS, and KOA. Second, while the chosen disease pair is biologically plausible, focusing on AS with KOA does not capture other co-occurring patterns. Third, participants were employees of a single, predominantly male industrial enterprise, and their demographic characteristics, work-related exposures, health status, and access to occupational health examinations may differ from those of workers in other industries or the general population. Therefore, the findings should not be generalized beyond similar occupational settings without external validation. Fourth, the inverse association in women with GDR ≥ 1 was based on only a few cases of co-occurrence in this subgroup, yielding wide confidence intervals and unstable estimates. Sex-specific power was limited, particularly among women, given the small number of cases in key subgroups. Fifth, dietary and lifestyle data were self-reported and subject to measurement error. Sixth, multiple testing across dietary indicators, scoring forms, and subgroups was not adjusted for, increasing the risk of type I error; the significant finding in women should be viewed as exploratory pending replication. Seventh, the DQQ captures dietary intake over a single 24-h period, which may not reflect habitual long term dietary patterns relevant to chronic disease outcomes, potentially attenuating associations. Eighth, we cannot rule out residual confounding from unmeasured factors, including educational attainment, income, occupational subtype, shift work, job-specific physical workload, total energy intake, medication use, and family history of chronic disease. In particular, working hours, shift pattens, and physical workload may be associated with both dietary behaviors and the development of AS and KOA. The observed associations may therefore have been either attenuated or inflated due to residual cofounding. Future prospective occupational studies should collect detailed work-related exposure data and assess their potential confounding or effect-modifying roles. Finally, biases inherent to occupational epidemiology, including collider bias, healthy worker effect, and survivor bias, may influence results. Hypertension and diabetes were not included as covariates in the primary model; however, sensitivity analyses excluding participants with both conditions and separately excluding participants with hypertension or diabetes were performed and were generally consistent with the primary findings. These sensitivity analyses reduce, but do not eliminate, concern about residual confounding because disease-history information and exposure ascertainment may be imperfect.

## 5. Conclusions

Higher diet quality, characterized by greater adherence to global dietary recommendations, was inversely associated with the co-occurrence of AS and KOA among female workers; however, this finding should be considered exploratory and interpreted cautiously because of the small number of women and the limited number of co-occurrence cases in the highest GDR category. Further prospective studies with larger and more representative occupational populations are needed to confirm this association.

## Figures and Tables

**Table 1:** Characteristics of participants, categorized by gender and AS/KOA status.

Characteristics	Reference (*n* = 182)	AS (*n* = 359)	KOA (*n* = 220)	Co-Occurrence (*n* = 579)	*p* Value
Men (*n*)	182	359	220	579	
Age, years, median (IQR)	38 (9)	40 (11)	39 (9)	41 (12)	<0.001
Age, *n* (%)					0.003
21–40 years	114 (62.64)	191 (53.20)	126 (57.27)	278 (48.01)	
≥41 years	68 (37.36)	168 (46.80)	94 (42.73)	301 (51.99)	
BMI, kg/m^2^, mean (SD)	25.59 (3.79)	25.95 (3.19)	26.13 (3.29)	26.42 (3.77)	0.03
SBP, mmHg, mean (SD)	125.91 (12.00)	134.29 (13.54)	127.02 (11.07)	135.85 (14.67)	<0.001
DBP, mmHg, mean (SD)	79.45 (8.66)	84.69 (10.12)	79.88 (8.92)	85.30 (10.36)	<0.001
Ethnicity, *n* (%)					<0.001
Han	174 (95.60)	343 (95.54)	200 (90.91)	566 (97.75)	
Other	8 (4.40)	16 (4.46)	20 (9.09)	13 (2.25)	
Smoking status, *n* (%) *					0.798
Never smoking	89 (48.90)	165 (45.96)	102 (46.36)	273 (47.23)	
Quit smoking	8 (4.40)	25 (6.96)	15 (6.82)	35 (6.06)	
Occasional smoking	18 (9.89)	23 (6.41)	19 (8.64)	38 (6.57)	
Regular smoking	67 (36.81)	146 (40.67)	84 (38.18)	232 (40.14)	
Drinking status, *n* (%)					0.097
Never drinking	43 (23.63)	100 (27.86)	60 (27.27)	184 (31.78)	
Quit drinking	2 (1.10)	3 (0.84)	5 (2.27)	9 (1.55)	
Occasional drinking	119 (65.38)	206 (57.38)	132 (60.00)	302 (52.16)	
Regular drinking	18 (9.89)	50 (13.93)	23 (10.45)	84 (14.51)	
Physical activity, *n* (%)					0.755
Regular exercise	79 (43.41)	139 (38.72)	90 (40.91)	260 (44.91)	
Occasional exercise	51 (28.02)	120 (33.43)	70 (31.82)	163 (28.15)	
Some exercise	17 (9.34)	34 (9.47)	18 (8.18)	45 (7.77)	
None	35 (19.23)	66 (18.38)	42 (19.09)	111 (19.17)	
Diet quality indicators, median (IQR)					
NCD-Protect	6 (5)	5 (6)	5 (6)	5 (5)	0.039
NCD-Risk	7 (4)	7 (6)	6 (5)	7 (6)	0.094
GDR	−1 (2)	−1 (2)	−1 (3)	−1 (2)	0.836
DDS	20 (10)	20 (16)	18 (15)	19 (12)	0.053
Hypertension, *n* (%)	32 (17.58)	132 (36.77)	38 (17.27)	252 (43.52)	<0.001
Diabetes, *n* (%)	6 (3.30)	16 (4.46)	6 (2.73)	26 (4.49)	0.634
Hyperlipidemia, *n* (%)	7 (3.85)	22 (6.13)	6 (2.73)	25 (4.32)	0.255
Women (*n*)	35	25	90	48	
Age, years, median (IQR)	38 (6)	38 (5)	39 (4)	41 (7)	<0.001
Age, *n* (%)					0.067
21–40 years	25 (71.43)	18 (72.00)	57 (63.33)	23 (47.92)	
≥41 years	10 (28.57)	7 (28.00)	33 (36.67)	25 (52.08)	
BMI, kg/m^2^, mean (SD)	23.97 (3.47)	24.96 (4.77)	22.86 (3.07)	25.28 (3.14)	<0.001
SBP, mmHg, mean (SD)	120.40 (10.97)	122.64 (11.72)	120.68 (11.80)	133.06 (14.64)	<0.001
DBP, mmHg, mean (SD)	74.09 (7.91)	76.56 (8.95)	73.64 (9.27)	82.15 (11.34)	<0.001
Ethnicity, *n* (%)					0.553
Han	32 (91.43)	24 (96.00)	84 (93.33)	47 (97.92)	
Other	3 (8.57)	1 (4.00)	6 (6.67)	1 (2.08)	
Smoking status, *n* (%) *					0.408
Never smoking	35 (100.00)	25 (100.00)	90 (100.00)	46 (95.83)	
Quit smoking	0 (0.00)	0 (0.00)	0 (0.00)	0 (0.00)	
Occasional smoking	0 (0.00)	0 (0.00)	0 (0.00)	1 (2.08)	
Regular smoking	0 (0.00)	0 (0.00)	0 (0.00)	1 (2.08)	
Drinking status, *n* (%)					0.969
Never drinking	33 (94.29)	24 (96.00)	84 (93.33)	46 (95.83)	
Quit drinking	0 (0.00)	0 (0.00)	0 (0.00)	0 (0.00)	
Occasional drinking	2 (5.71)	1 (4.00)	6 (6.67)	2 (4.17)	
Regular drinking	0 (0.00)	0 (0.00)	0 (0.00)	0 (0.00)	
Physical activity, *n* (%)					0.443
Regular exercise	7 (20.00)	7 (28.00)	32 (35.56)	13 (27.08)	
Occasional exercise	11 (31.43)	7 (28.00)	31 (34.44)	14 (29.17)	
Some exercise	0 (0.00)	1 (4.00)	2 (2.22)	3 (6.25)	
None	17 (48.57)	10 (40.00)	25 (27.78)	18 (37.50)	
Diet quality indicators, median (IQR)					
NCD-Protect	5 (4)	6 (5)	6 (6)	5 (4)	0.862
NCD-Risk	7 (4)	7 (4)	7 (4)	8 (3)	0.433
GDR	0 (3)	0 (3)	−1 (2)	−2 (2)	0.018
DDS	20 (8)	21 (12)	20 (10)	19 (8)	0.804
Hypertension, *n* (%)	3 (8.57)	3 (12.00)	4 (4.44)	13 (27.08)	<0.001
Diabetes, *n* (%)	1 (2.86)	1 (4.00)	1 (1.11)	1 (2.08)	0.801
Hyperlipidemia, *n* (%)	1 (2.86)	0 (0.00)	0 (0.00)	1 (2.08)	0.407

Note: * *n* = 1537 for smoking status; *p* values were calculated using *t* test, Wilcoxon rank-sum, or χ^2^, as appropriate; IQR = interquartile range; SD = standard deviation; BMI = body mass index; SBP = systolic blood pressure; DBP = diastolic blood pressure; NCD = non-communicable disease; GDR = global dietary recommendations.

**Table 2:** Number and percent (%) of co-occurrence cases by categories of diet quality indicators.

	Total (*n* = 1538)	Men (*n* = 1340)	Women (*n* = 198)
Total co-occurrence cases	627/1538 (40.77)	579/1340 (43.21)	48/198 (24.24)
NCD-Protect score			
≤3	216/533 (40.53)	200/472 (42.37)	16/61 (26.23)
4~6	188/451 (41.69)	169/384 (44.01)	19/67 (28.36)
≥7	223/554 (40.25)	210/484 (43.39)	13/70 (18.57)
NCD-Risk score			
≤4	176/431 (40.84)	169/387 (43.67)	7/44 (15.91)
5~8	267/675 (39.56)	245/582 (42.10)	22/93 (23.66)
9	184/432 (42.59)	165/371 (44.47)	19/61 (31.15)
GDR score			
≤−3	153/378 (40.48)	133/318 (41.82)	20/60 (33.33)
−2~0	359/905 (39.67)	334/792 (42.17)	25/113 (22.12)
≥1	115/255 (45.10)	112/230 (48.70)	3/25 (12.00)
DDS			
≤10	152/387 (39.28)	144/347 (41.50)	8/40 (20.00)
11~20	206/465 (44.30)	182/393 (46.31)	24/72 (33.33)
≥21	269/686 (39.21)	253/600 (42.17)	16/86 (18.60)

Note: NCD = non-communicable disease; GDR = global dietary recommendations; DDS = dietary diversity score.

**Table 3:** Association between continuous diet quality indicators and comorbidities.

Diet Quality Indicators	Total (*n* = 1538)		Men (*n* = 1340)		Women (*n* = 198)	
	OR (95% CI)	*p* Value	OR (95% CI)	*p* Value	OR (95% CI)	*p* Value
NCD-Protect score	1.00 (0.96~1.03)	0.845	1.00 (0.97~1.04)	0.877	0.90 (0.78~1.02)	0.107
NCD-Risk score	1.00 (0.97~1.03)	0.930	0.99 (0.96~1.03)	0.677	1.04 (0.91~1.19)	0.544
GDR score	1.00 (0.95~1.05)	0.881	1.02 (0.97~1.08)	0.388	0.75 (0.62~0.91)	0.003 *
Dietary Diversity Score	1.00 (0.99~1.01)	0.638	1.00 (0.99~1.01)	0.735	0.98 (0.93~1.02)	0.349

Note: * *p* < 0.05. NCD = non-communicable disease; GDR = global dietary recommendations. OR = odds ratios; CI = confidence intervals. The total-sample models were adjusted for sex, age, ethnicity, BMI, SBP, DBP, smoking, drinking, and physical activity. Sex-stratified models were adjusted for the same covariates except sex. The multivariable regression analyses were based on complete cases. Participants with missing data for the dietary indicator, AS/KOA co-occurrence status, or any covariate included in the corresponding model were excluded. Consequently, the analytical sample sizes and case counts may differ slightly from the descriptive results presented in Table 2.

**Table 4:** Association between categorical diet quality indicators and comorbidities.

Diet Quality Indicators	Total (*n* = 1538)			Men (*n* = 1340)			Women (*n* = 198)		
	Case	OR (95% CI)	*p* Value	Case	OR (95% CI)	*p* Value	Case	OR (95% CI)	*p* Value
NCD-Protect score									
≤3	216	1.00 (reference)	-	200	1.00 (reference)	-	16	1.00 (reference)	-
4~6	188	1.05 (0.81~1.37)	0.713	169	1.05 (0.79~1.39)	0.737	19	0.64 (0.25~1.62)	0.345
≥7	223	0.95 (0.73~1.22)	0.677	210	1.01 (0.77~1.32)	0.948	13	0.39 (0.14~1.03)	0.057
NCD-Risk score									
≤4	176	1.00 (reference)	-	169	1.00 (reference)	-	7	1.00 (reference)	-
5~8	267	0.94 (0.72~1.21)	0.615	245	0.91 (0.69~1.19)	0.484	22	1.08 (0.39~3.03)	0.877
9	184	1.02 (0.77~1.35)	0.915	165	0.96 (0.72~1.29)	0.801	19	1.47 (0.50~4.30)	0.481
GDR score									
≤−3	153	1.00 (reference)	-	133	1.00 (reference)	-	20	1.00 (reference)	-
−2~0	359	0.96 (0.74~1.25)	0.773	334	1.04 (0.79~1.37)	0.766	25	0.60 (0.26~1.38)	0.228
≥1	115	1.16 (0.83~1.63)	0.395	112	1.35 (0.94~1.92)	0.102	3	0.13 (0.02~0.77)	0.025 *
Dietary Diversity Score									
≤10	152	1.00 (reference)	-	144	1.00 (reference)	-	8	1.00 (reference)	-
11~20	206	1.24 (0.93~1.66)	0.138	182	1.19 (0.88~1.61)	0.259	24	1.17 (0.42~3.25)	0.768
≥21	269	0.94 (0.72~1.23)	0.673	253	0.97 (0.73~1.28)	0.826	16	0.56 (0.19~1.60)	0.280

Note: * *p* < 0.05. NCD = non-communicable disease; GDR = global dietary recommendations; OR = odds ratios; CI = confidence intervals. The total-sample models were adjusted for sex, age, ethnicity, BMI, SBP, DBP, smoking, drinking, and physical activity. Sex-stratified models were adjusted for the same covariates except sex. The multivariable regression analyses were based on complete cases. Participants with missing data for the dietary indicator, AS/KOA co-occurrence status, or any covariate included in the corresponding model were excluded.

**Table 5:** Sensitivity analysis after excluding participants with both hypertension and diabetes using Firth penalized logistic regression.

Diet Quality Indicators	Total (*n* = 1242)			Men (*n* = 1054)			Women (*n* = 188)		
	Case	OR (95% CI)	*p* Value	Case	OR (95% CI)	*p* Value	Case	OR (95% CI)	*p* Value
NCD-Protect score									
Continuous		1.00 (0.96~1.04)	0.893		1.01 (0.97~1.05)	0.778		0.91 (0.80~1.03)	0.145
Categories									
≤3	164	1.00 (reference)	-	150	1.00 (reference)	-	14	1.00 (reference)	-
4~6	136	1.03 (0.76~1.38)	0.867	119	1.02 (0.74~1.40)	0.906	17	0.72 (0.29~1.79)	0.485
≥7	165	0.93 (0.70~1.24)	0.633	154	1.00 (0.74~1.35)	0.986	11	0.44 (0.16~1.14)	0.090
NCD-Risk score									
Continuous		0.99 (0.96~1.03)	0.759		0.99 (0.95~1.02)	0.470		1.05 (0.93~1.21)	0.428
Categories									
≤4	137	1.00 (reference)	-	130	1.00 (reference)	-	7	1.00 (reference)	-
5~8	192	0.87 (0.65~1.16)	0.349	175	0.85 (0.63~1.15)	0.306	17	0.99 (0.37~2.73)	0.977
9	136	1.03 (0.75~1.42)	0.840	118	0.97 (0.69~1.35)	0.842	18	1.48 (0.54~4.29)	0.447
GDR score									
Continuous		1.01 (0.95~1.07)	0.791		1.05 (0.99~1.11)	0.130		0.75 (0.62~0.90)	0.001 *
Categories									
≤−3	105	1.00 (reference)	-	87	1.00 (reference)	-	18	1.00 (reference)	-
−2~0	273	1.01 (0.76~1.36)	0.927	250	1.13 (0.83~1.55)	0.442	23	0.59 (0.26~1.34)	0.205
≥1	87	1.28 (0.87~1.87)	0.211	86	1.59 (1.06~2.39)	0.024 *	1	0.12 (0.01~0.63)	0.009 *
Dietary Diversity Score									
Continuous		1.00 (0.98~1.01)	0.521		1.00 (0.98~1.01)	0.587		0.98 (0.94~1.03)	0.472
Categories									
≤10	118	1.00 (reference)	-	110	1.00 (reference)	-	8	1.00 (reference)	-
11~20	149	1.10 (0.80~1.51)	0.557	130	1.06 (0.76~1.49)	0.728	19	1.07 (0.41~2.93)	0.885
≥21	198	0.91 (0.67~1.23)	0.532	183	0.94 (0.69~1.29)	0.700	15	0.58 (0.21~1.62)	0.292

Note: * *p* < 0.05. NCD = non-communicable disease; GDR = global dietary recommendations. All odds ratios (OR) and 95% confidence intervals (CI) were estimated after adjusting for age, ethnicity, BMI, SBP, DBP, smoking, drinking, and physical activity.

## Data Availability

The data presented in this study are available on request from the corresponding author due to privacy and ethical restrictions. The data are not publicly available as they contain information that could compromise the privacy of research participants.

## Supplementary Materials

The following supporting information can be downloaded at https://www.mdpi.com/article/10.3390/nu18203352/s1, Table S1: Characteristics of participants, categorized by sex; Table S2: Firth-corrected associations between diet quality indicators and the co-occurrence of arteriosclerosis and knee osteoarthritis; Table S3: Firth-corrected associations between diet quality indicators and the co-occurrence of arteriosclerosis and knee osteoarthritis with FDR adjustment; Table S4: Associations between diet quality indicators and the co-occurrence of arteriosclerosis and knee osteoarthritis with FDR adjustment; Table S5: Sensitivity analysis of the association between diet quality indicators and the co-occurrence of arteriosclerosis (AS) and knee osteoarthritis (KOA) using a prespecified parsimonious model among women; Table S6: Sensitivity analysis after excluding participants with hypertension using Firth penalized logistic regression; Table S7: Sensitivity analysis after excluding participants with diabetes using Firth penalized logistic regression; Table S8: Sensitivity analysis using SBP-only adjustment with Firth penalized logistic regression; Table S9: Sensitivity analysis using DBP-only adjustment with Firth penalized logistic regression; Table S10: Sensitivity analysis without adjustment for SBP or DBP using Firth penalized logistic regression; Table S11: Associations of diet quality indicators with AS, KOA, and AS + KOA relative to the Reference group in the total sample; Table S12: Associations of diet quality indicators with AS, KOA, and AS + KOA relative to the Reference group among men; Table S13: Associations of diet quality indicators with AS, KOA, and AS + KOA relative to the Reference group among women; Table S14: Sensitivity analysis with additional adjustment for hyperlipidemia and elevated LDL-C using Firth penalized logistic regression; Figure S1: Distribution of DQQ-derived diet quality scores among 1538 participants. Bars show the number of participants at each integer score. Dotted vertical lines indicate the category boundaries used in the categorical analyses.
