# Supplementary material for: Diet Quality and Co-Occurrence of Arteriosclerosis and Knee Osteoarthritis: A Cross-Sectional Survey Among Chinese Iron Workers from Shougang Group
Source: Nutrients. 2026 Oct 13;18(20):3352. doi: 10.3390/nu18203352 (PMC13708492; doi:10.3390/nu18203352)

Supplementary materials

Supplementary Table S1. Characteristics of participants, categorized by sex.

Characteristics	Total (n=1538)	Men (n=1340)	Women (n=198)	t/Z/χ2 values	P values#
Age, years, median (IQR)	40 (10)	40 (11)	39 (5)	-2.50	0.012
Age, n (%)					0.019
21-40 years	832 (54.10)	709 (52.91)	123 (62.12)		
⩾41 years	706 (45.90)	631 (47.09)	75 (37.88)		
BMI, kg/m2, mean (SD)	25.85 (3.63)	26.13 (3.56)	23.91 (3.54)	8.24	<0.001
SBP, mmHg, mean (SD)	131.51 (14.28)	132.63 (14.07)	123.88 (13.38)	8.07	<0.001
DBP, mmHg, mean (SD)	82.51 (10.43)	83.45 (10.15)	76.15 (10.12)	9.34	<0.001
Ethnicity, n (%)					0.416
Han	1470 (95.58)	1283 (95.75)	187 (94.44)		
Other	68 (4.42)	57 (4.25)	11 (5.56)		
Smoking status, n (%)*					<0.001
Never smoking	825 (53.68)	629 (46.98)	196 (98.99)		
Quit smoking	83 (5.40)	83 (6.20)	0 (0.00)		
Occasional smoking	99 (6.44)	98 (7.32)	1 (0.51)		
Regular smoking	530 (34.48)	529 (39.51)	1 (0.51)		
Drinking status, n (%)					<0.001
Never drinking	574 (37.32)	387 (28.88)	187 (94.44)		
Quit drinking	19 (1.24)	19 (1.42)	0 (0.00)		
Occasional drinking	770 (50.07)	759 (56.64)	11 (5.56)		
Regular drinking	175 (11.38)	175 (13.06)	0 (0.00)		
Physical activity, n (%)					<0.001
Regular exercise	627 (40.77)	568 (42.39)	59 (29.80)		
Occasional exercise	467 (30.36)	404 (30.15)	63 (31.82)		
Some exercise	120 (7.80)	114 (8.51)	6 (3.03)		
None	324 (21.07)	254 (18.96)	70 (35.35)		
Diet quality indicators, median (IQR)					
NCD-Protect	5 (5)	5 (5)	6 (4)	0.04	0.967
NCD-Risk	7 (5)	7 (6)	7 (4)	1.91	0.056
GDR	-1 (2)	-1 (2)	-1 (3)	-2.58	0.01
DDS	19 (13)	19 (13)	19 (9)	0.379	0.705
Arteriosclerosis	1011 (65.73)	938 (70.00)	73 (36.87)	82.61	<0.001
Knee osteoarthritis	937 (60.92)	799 (59.63)	138 (69.70)	6.93	0.008
Hypertension	477 (31.01)	454 (33.88)	23 (11.62)	38.38	<0.001
Diabetes	58 (3.77)	54 (4.03)	4 (2.02)	1.95	0.162
Hyperlipidemia	62 (4.03)	60 (4.48)	2 (1.01)	5.41	0.020

Supplementary Table S2. Firth-corrected associations between diet quality indicators and the co-occurrence of arteriosclerosis and knee osteoarthritis.

Diet quality indicators	Total		Men		Women	
	OR (95% CI)	P value	OR (95% CI)	P value	OR (95% CI)	P value
NCD-Protect score						
Continuous	1.00 (0.96~1.03)	0.839	1.00 (0.97~1.04)	0.870	0.90 (0.79~1.03)	0.125
Categories						
≤3	1.00 (reference)	-	1.00 (reference)	-	1.00 (reference)	-
4~6	1.05 (0.81~1.37)	0.716	1.05 (0.79~1.39)	0.741	0.66 (0.27~1.61)	0.364
≥7	0.95 (0.74~1.22)	0.679	1.01 (0.77~1.31)	0.949	0.42 (0.16~1.06)	0.065
NCD-Risk score						
Continuous	1.00 (0.97~1.03)	0.924	0.99 (0.96~1.03)	0.673	1.04 (0.92~1.19)	0.547
Categories						
≤4	1.00 (reference)	-	1.00 (reference)	-	1.00 (reference)	-
5~8	0.94 (0.72~1.21)	0.613	0.91 (0.70~1.19)	0.483	1.08 (0.41~2.98)	0.882
9	1.02 (0.77~1.35)	0.917	0.96 (0.72~1.29)	0.799	1.45 (0.53~4.23)	0.473
GDR score						
Continuous	1.00 (0.95~1.05)	0.877	1.02 (0.97~1.08)	0.378	0.76 (0.63~0.91)	0.003*
Categories						
≤-3	1.00 (reference)	-	1.00 (reference)	-	1.00 (reference)	-
-2~0	0.96 (0.75~1.24)	0.771	1.04 (0.79~1.37)	0.770	0.61 (0.27~1.38)	0.233
≥1	1.16 (0.83~1.62)	0.396	1.34 (0.94~1.91)	0.102	0.18 (0.03~0.75)	0.018*
Dietary Diversity Score						
Continuous	1.00 (0.99~1.01)	0.634	1.00 (0.99~1.01)	0.732	0.98 (0.94~1.03)	0.375
Categories						
≤10	1.00 (reference)	-	1.00 (reference)	-	1.00 (reference)	-
11~20	1.24 (0.93~1.65)	0.139	1.19 (0.88~1.60)	0.262	1.16 (0.44~3.14)	0.771
≥21	0.94 (0.72~1.23)	0.672	0.97 (0.74~1.28)	0.823	0.58 (0.21~1.64)	0.301

Firth penalized logistic regression models adjusted for sex, age, ethnicity, BMI, SBP, DBP, smoking, drinking, and physical activity.

Supplementary Table S3. Firth-corrected associations between diet quality indicators and the co-occurrence of arteriosclerosis and knee osteoarthritis with FDR adjustment

Diet quality indicators	Total		Men		Women	
	OR (95% CI)	P value	OR (95% CI)	P value	OR (95% CI)	P value
NCD-Protect score						
Continuous	1.00 (0.96~1.03)	0.928	1.00 (0.97~1.04)	0.928	0.90 (0.79~1.03)	0.825
Categories						
≤3	1.00 (reference)	-	1.00 (reference)	-	1.00 (reference)	-
4~6	1.05 (0.81~1.37)	0.949	1.05 (0.79~1.39)	0.949	0.66 (0.27~1.61)	0.949
≥7	0.95 (0.74~1.22)	0.949	1.01 (0.77~1.31)	0.949	0.42 (0.16~1.06)	0.779
NCD-Risk score						
Continuous	1.00 (0.97~1.03)	0.928	0.99 (0.96~1.03)	0.928	1.04 (0.92~1.19)	0.928
Categories						
≤4	1.00 (reference)	-	1.00 (reference)	-	1.00 (reference)	-
5~8	0.94 (0.72~1.21)	0.949	0.91 (0.70~1.19)	0.949	1.08 (0.41~2.98)	0.949
9	1.02 (0.77~1.35)	0.949	0.96 (0.72~1.29)	0.949	1.45 (0.53~4.23)	0.949
GDR score						
Continuous	1.00 (0.95~1.05)	0.928	1.02 (0.97~1.08)	0.928	0.76 (0.63~0.91)	0.100
Categories						
≤-3	1.00 (reference)	-	1.00 (reference)	-	1.00 (reference)	-
-2~0	0.96 (0.75~1.24)	0.949	1.04 (0.79~1.37)	0.949	0.61 (0.27~1.38)	0.949
≥1	1.16 (0.83~1.62)	0.949	1.34 (0.94~1.91)	0.835	0.18 (0.03~0.75)	0.316
Dietary Diversity Score						
Continuous	1.00 (0.99~1.01)	0.928	1.00 (0.99~1.01)	0.928	0.98 (0.94~1.03)	0.928
Categories						
≤10	1.00 (reference)	-	1.00 (reference)	-	1.00 (reference)	-
11~20	1.24 (0.93~1.65)	0.835	1.19 (0.88~1.60)	0.949	1.16 (0.44~3.14)	0.949
≥21	0.94 (0.72~1.23)	0.949	0.97 (0.74~1.28)	0.949	0.58 (0.21~1.64)	0.949

Supplementary Table S4. Associations between diet quality indicators and the co-occurrence of arteriosclerosis and knee osteoarthritis with FDR adjustment

Diet quality indicators	Total		Men		Women	
	OR (95% CI)	P value	OR (95% CI)	P value	OR (95% CI)	P value
NCD-Protect score						
Continuous	1.00 (0.96~1.03)	0.928	1.00 (0.97~1.04)	0.928	0.90 (0.78~1.02)	0.770
Categories						
≤3	1.00 (reference)	-	1.00 (reference)	-	1.00 (reference)	-
4~6	1.05 (0.81~1.37)	0.948	1.05 (0.79~1.39)	0.948	0.64 (0.25~1.62)	0.948
≥7	0.95 (0.73~1.22)	0.948	1.01 (0.77~1.32)	0.948	0.39 (0.14~1.03)	0.678
NCD-Risk score						
Continuous	1.00 (0.97~1.03)	0.928	0.99 (0.96~1.03)	0.928	1.04 (0.92~1.20)	0.928
Categories						
≤4	1.00 (reference)	-	1.00 (reference)	-	1.00 (reference)	-
5~8	0.94 (0.72~1.21)	0.948	0.91 (0.69~1.19)	0.948	1.08 (0.39~3.03)	0.948
9	1.02 (0.77~1.35)	0.948	0.96 (0.72~1.29)	0.948	1.47 (0.50~4.30)	0.948
GDR score						
Continuous	1.00 (0.95~1.05)	0.928	1.02 (0.97~1.08)	0.928	0.75 (0.62~0.90)	0.107
Categories						
≤-3	1.00 (reference)	-	1.00 (reference)	-	1.00 (reference)	-
-2~0	0.96 (0.74~1.25)	0.948	1.04 (0.79~1.37)	0.948	0.60 (0.26~1.38)	0.948
≥1	1.16 (0.83~1.63)	0.948	1.35 (0.94~1.92)	0.77	0.13 (0.02~0.77)	0.443
Dietary Diversity Score						
Continuous	1.00 (0.99~1.01)	0.928	1.00 (0.99~1.01)	0.928	0.98 (0.93~1.03)	0.928
Categories						
≤10	1.00 (reference)	-	1.00 (reference)	-	1.00 (reference)	-
11~20	1.24 (0.93~1.66)	0.825	1.19 (0.88~1.61)	0.948	1.17 (0.42~3.25)	0.948
≥21	0.94 (0.72~1.23)	0.948	0.97 (0.73~1.28)	0.948	0.56 (0.19~1.60)	0.948

Supplementary Table S5. Sensitivity analysis of the association between diet quality indicators and the co-occurrence of arteriosclerosis (AS) and knee osteoarthritis (KOA) using a prespecified parsimonious model among women.

Diet quality indicators	Women(n=198)	
	OR (95% CI)	P value
NCD-Protect score		
Continuous	0.92 (0.81~1.05)	0.212
Categories	-	-
≤3	1.00 (reference)	-
4~6	0.68 (0.28~1.65)	0.398
≥7	0.41 (0.15~1.03)	0.059
NCD-Risk score		
Continuous	1.06 (0.93~1.21)	0.394
Categories	-	-
≤4	1.00 (reference)	-
5~8	1.11 (0.43~3.06)	0.832
9	1.45 (0.53~4.21)	0.474
GDR score		
Continuous	0.77 (0.64~0.91)	0.003*
Categories	-	-
≤-3	1.00 (reference)	-
-2~0	0.58 (0.26~1.30)	0.184
≥1	0.17 (0.03~0.74)	0.016*
No. of DQQ food groups		
Continuous	0.99 (0.94~1.03)	0.504
Categories	-	-
≤10	1.00 (reference)	-
11~20	1.19 (0.45~3.22)	0.727
≥21	0.58 (0.21~1.61)	0.287

Supplementary Table S6. Sensitivity analysis after excluding participants with hypertension using Firth penalized logistic regression.

Diet quality indicators	Total		Men		Women	
	**OR (95% CI)**	**P value**	**OR (95% CI)**	**P value**	**OR (95% CI)**	**P value**
NCD-Protect score						
Continuous	0.99 (0.95~1.03)	0.670	1.00 (0.96~1.04)	0.954	0.92 (0.80~1.04)	0.177
Categories						
≤3	1.00 (reference)	-	1.00 (reference)	-	1.00 (reference)	-
4~6	1.04 (0.77~1.39)	0.813	1.03 (0.75~1.41)	0.851	0.75 (0.30~1.83)	0.523
≥7	0.91 (0.68~1.20)	0.489	0.96 (0.72~1.29)	0.808	0.46 (0.17~1.18)	0.107
NCD-Risk score						
Continuous	0.99 (0.96~1.03)	0.600	0.98 (0.95~1.02)	0.343	1.05 (0.93~1.21)	0.416
Categories						
≤4	1.00 (reference)	-	1.00 (reference)	-	1.00 (reference)	-
5~8	0.87 (0.65~1.15)	0.322	0.85 (0.63~1.14)	0.275	1.02 (0.39~2.82)	0.968
9	1.01 (0.74~1.39)	0.929	0.95 (0.68~1.31)	0.737	1.55 (0.57~4.47)	0.397
GDR score						
Continuous	1.00 (0.95~1.06)	0.861	1.04 (0.98~1.10)	0.171	0.75 (0.62~0.90)	0.002*
Categories						
≤-3	1.00 (reference)	-	1.00 (reference)	-	1.00 (reference)	-
-2~0	1.01 (0.76~1.35)	0.925	1.11 (0.82~1.52)	0.488	0.64 (0.28~1.44)	0.274
≥1	1.24 (0.85~1.81)	0.263	1.51 (1.02~2.25)	0.040*	0.13 (0.01~0.69)	0.013*
No. of DQQ food groups						
Continuous	0.99 (0.98~1.01)	0.405	0.99 (0.98~1.01)	0.448	0.98 (0.94~1.03)	0.503
Categories						
≤10	1.00 (reference)	-	1.00 (reference)	-	1.00 (reference)	-
11~20	1.12 (0.82~1.53)	0.477	1.08 (0.78~1.51)	0.635	1.12 (0.43~3.02)	0.825
≥21	0.89 (0.66~1.20)	0.439	0.91 (0.67~1.24)	0.565	0.61 (0.23~1.71)	0.342

Supplementary Table S7. Sensitivity analysis after excluding participants with diabetes using Firth penalized logistic regression.

Diet quality indicators	Total		Men		Women	
	**OR (95% CI)**	**P value**	**OR (95% CI)**	**P value**	**OR (95% CI)**	**P value**
NCD-Protect score						
Continuous	1.00 (0.97~1.04)	0.983	1.01 (0.97~1.05)	0.657	0.90 (0.78~1.02)	0.100
Categories						
≤3	1.00 (reference)	-	1.00 (reference)	-	1.00 (reference)	-
4~6	1.00 (0.77~1.32)	0.977	1.00 (0.75~1.33)	0.984	0.64 (0.26~1.57)	0.331
≥7	0.97 (0.75~1.26)	0.848	1.05 (0.80~1.37)	0.742	0.40 (0.15~1.01)	0.052
NCD-Risk score						
Continuous	1.00 (0.97~1.03)	0.983	0.99 (0.96~1.03)	0.752	1.04 (0.92~1.19)	0.562
Categories						
≤4	1.00 (reference)	-	1.00 (reference)	-	1.00 (reference)	-
5~8	0.94 (0.72~1.22)	0.616	0.91 (0.69~1.20)	0.509	1.03 (0.39~2.88)	0.946
9	1.02 (0.77~1.36)	0.886	0.97 (0.72~1.31)	0.841	1.38 (0.50~4.03)	0.540
GDR score						
Continuous	1.00 (0.95~1.05)	0.994	1.03 (0.98~1.09)	0.258	0.75 (0.62~0.90)	0.002*
Categories						
≤-3	1.00 (reference)	-	1.00 (reference)	-	1.00 (reference)	-
-2~0	0.97 (0.75~1.26)	0.816	1.06 (0.81~1.41)	0.660	0.56 (0.25~1.28)	0.168
≥1	1.19 (0.84~1.68)	0.327	1.42 (0.99~2.04)	0.059	0.16 (0.02~0.69)	0.012*
No. of DQQ food groups						
Continuous	1.00 (0.99~1.01)	0.717	1.00 (0.99~1.01)	0.834	0.98 (0.94~1.02)	0.346
Categories						
≤10	1.00 (reference)	-	1.00 (reference)	-	1.00 (reference)	-
11~20	1.20 (0.90~1.61)	0.219	1.15 (0.85~1.56)	0.373	1.11 (0.42~3.04)	0.837
≥21	0.95 (0.73~1.25)	0.724	0.98 (0.74~1.30)	0.903	0.55 (0.20~1.54)	0.247

Supplementary Table S8. Sensitivity analysis using SBP-only adjustment with Firth penalized logistic regression.

Diet quality indicators	Total		Men		Women	
	**OR (95% CI)**	**P value**	**OR (95% CI)**	**P value**	**OR (95% CI)**	**P value**
NCD-Protect score						
Continuous	1.00 (0.96~1.03)	0.839	1.00 (0.97~1.04)	0.872	0.90 (0.79~1.03)	0.120
Categories						
≤3	1.00 (reference)	-	1.00 (reference)	-	1.00 (reference)	-
4~6	1.05 (0.81~1.37)	0.716	1.05 (0.79~1.39)	0.740	0.68 (0.28~1.65)	0.398
≥7	0.95 (0.74~1.22)	0.678	1.01 (0.77~1.31)	0.948	0.41 (0.15~1.03)	0.059
NCD-Risk score						
Continuous	1.00 (0.97~1.03)	0.923	0.99 (0.96~1.03)	0.673	1.04 (0.92~1.19)	0.546
Categories						
≤4	1.00 (reference)	-	1.00 (reference)	-	1.00 (reference)	-
5~8	0.94 (0.72~1.21)	0.613	0.91 (0.70~1.19)	0.484	1.11 (0.43~3.06)	0.832
9	1.02 (0.77~1.35)	0.917	0.96 (0.72~1.29)	0.801	1.45 (0.53~4.21)	0.474
GDR score						
Continuous	1.00 (0.95~1.05)	0.879	1.02 (0.97~1.08)	0.382	0.76 (0.63~0.91)	0.002*
Categories						
≤-3	1.00 (reference)	-	1.00 (reference)	-	1.00 (reference)	-
-2~0	0.96 (0.75~1.24)	0.771	1.04 (0.79~1.37)	0.769	0.58 (0.26~1.30)	0.184
≥1	1.16 (0.83~1.62)	0.395	1.34 (0.94~1.91)	0.105	0.17 (0.03~0.74)	0.016*
Dietary Diversity Score						
Continuous	1.00 (0.99~1.01)	0.634	1.00 (0.99~1.01)	0.733	0.98 (0.94~1.02)	0.353
Categories						
≤10	1.00 (reference)	-	1.00 (reference)	-	1.00 (reference)	-
11~20	1.24 (0.93~1.65)	0.139	1.19 (0.88~1.60)	0.260	1.19 (0.45~3.22)	0.727
≥21	0.94 (0.72~1.23)	0.672	0.97 (0.74~1.28)	0.825	0.58 (0.21~1.61)	0.287

Supplementary Table S9. Sensitivity analysis using DBP-only adjustment with Firth penalized logistic regression.

Diet quality indicators	Total		Men		Women	
	OR (95% CI)	P value	OR (95% CI)	P value	OR (95% CI)	P value
NCD-Protect score						
Continuous	1.00 (0.96~1.03)	0.916	1.00 (0.97~1.04)	0.827	0.93 (0.82~1.05)	0.246
Categories						
≤3	1.00 (reference)	-	1.00 (reference)	-	1.00 (reference)	-
4~6	1.04 (0.80~1.35)	0.768	1.04 (0.79~1.38)	0.767	0.69 (0.28~1.66)	0.413
≥7	0.97 (0.75~1.25)	0.804	1.02 (0.79~1.33)	0.860	0.51 (0.20~1.25)	0.139
NCD-Risk score						
Continuous	1.00 (0.97~1.03)	0.977	0.99 (0.96~1.03)	0.703	1.05 (0.93~1.21)	0.405
Categories						
≤4	1.00 (reference)	-	1.00 (reference)	-	1.00 (reference)	-
5~8	0.93 (0.72~1.20)	0.584	0.91 (0.70~1.18)	0.469	1.14 (0.44~3.15)	0.785
9	1.03 (0.78~1.36)	0.845	0.97 (0.72~1.30)	0.844	1.60 (0.59~4.64)	0.360
GDR score						
Continuous	1.00 (0.95~1.05)	0.908	1.02 (0.97~1.08)	0.369	0.78 (0.66~0.93)	0.005*
Categories						
≤-3	1.00 (reference)	-	1.00 (reference)	-	1.00 (reference)	-
-2~0	0.98 (0.76~1.27)	0.883	1.05 (0.80~1.38)	0.709	0.69 (0.32~1.53)	0.358
≥1	1.16 (0.83~1.62)	0.384	1.34 (0.94~1.90)	0.106	0.22 (0.04~0.88)	0.032*
Dietary Diversity Score						
Continuous	1.00 (0.99~1.01)	0.722	1.00 (0.99~1.01)	0.791	0.99 (0.95~1.03)	0.589
Categories						
≤10	1.00 (reference)	-	1.00 (reference)	-	1.00 (reference)	-
11~20	1.23 (0.93~1.64)	0.152	1.18 (0.88~1.59)	0.275	1.25 (0.48~3.39)	0.644
≥21	0.96 (0.74~1.25)	0.773	0.98 (0.75~1.29)	0.904	0.68 (0.25~1.89)	0.452

.

Supplementary Table S10. Sensitivity analysis without adjustment for SBP or DBP using Firth penalized logistic regression.

Diet quality indicators	Total		Men		Women	
	**OR (95% CI)**	**P value**	**OR (95% CI)**	**P value**	**OR (95% CI)**	**P value**
NCD-Protect score						
Continuous	1.00 (0.97~1.04)	0.849	1.01 (0.97~1.04)	0.675	0.97 (0.86~1.09)	0.565
Categories						
≤3	1.00 (reference)	-	1.00 (reference)	-	1.00 (reference)	-
4~6	1.04 (0.80~1.35)	0.780	1.03 (0.78~1.35)	0.856	0.96 (0.42~2.18)	0.914
≥7	0.99 (0.78~1.27)	0.963	1.04 (0.80~1.35)	0.754	0.63 (0.26~1.49)	0.291
NCD-Risk score						
Continuous	1.00 (0.97~1.03)	0.953	0.99 (0.96~1.03)	0.702	1.09 (0.97~1.23)	0.169
Categories						
≤4	1.00 (reference)	-	1.00 (reference)	-	1.00 (reference)	-
5~8	0.93 (0.72~1.19)	0.555	0.89 (0.69~1.16)	0.399	1.46 (0.59~3.92)	0.415
9	1.04 (0.79~1.37)	0.777	0.97 (0.73~1.30)	0.853	1.98 (0.76~5.55)	0.163
GDR score						
Continuous	1.00 (0.96~1.05)	0.857	1.03 (0.98~1.09)	0.235	0.78 (0.66~0.93)	0.004*
Categories						
≤-3	1.00 (reference)	-	1.00 (reference)	-	1.00 (reference)	-
-2~0	0.99 (0.77~1.28)	0.959	1.07 (0.82~1.40)	0.611	0.61 (0.29~1.30)	0.198
≥1	1.22 (0.88~1.70)	0.231	1.41 (1.00~1.99)	0.053	0.21 (0.03~0.85)	0.028*
Dietary Diversity Score						
Continuous	1.00 (0.99~1.01)	0.808	1.00 (0.99~1.01)	0.826	1.00 (0.96~1.04)	0.933
Categories						
≤10	1.00 (reference)	-	1.00 (reference)	-	1.00 (reference)	-
11~20	1.21 (0.92~1.60)	0.181	1.14 (0.85~1.54)	0.372	1.70 (0.70~4.46)	0.248
≥21	0.97 (0.75~1.26)	0.813	0.98 (0.75~1.29)	0.912	0.81 (0.32~2.19)	0.672

Supplementary Table S11. Associations of diet quality indicators with AS, KOA, and AS+KOA relative to the Reference group in the total sample.

Diet quality indicators	AS		KOA		AS+KOA	
	**OR (95% CI)**	**P value**	**OR (95% CI)**	**P value**	**OR (95% CI)**	**P value**
NCD-Protect score						
Continuous	0.94 (0.89~0.99)	0.032*	0.94 (0.88~0.99)	0.024*	0.95 (0.90~1.00)	0.055
Categories						
≤3	1.00 (reference)	-	1.00 (reference)	-	1.00 (reference)	-
4~6	0.90 (0.57~1.41)	0.633	0.89 (0.57~1.41)	0.624	0.86 (0.56~1.32)	0.484
≥7	0.71 (0.46~1.10)	0.123	0.70 (0.46~1.06)	0.095	0.73 (0.49~1.09)	0.121
NCD-Risk score						
Continuous	0.96 (0.91~1.01)	0.142	0.95 (0.90~1.01)	0.092	0.96 (0.91~1.01)	0.111
Categories						
≤4	1.00 (reference)	-	1.00 (reference)	-	1.00 (reference)	-
5~8	0.84 (0.54~1.31)	0.445	0.79 (0.51~1.23)	0.306	0.78 (0.51~1.17)	0.231
9	0.60 (0.37~0.98)	0.039*	0.60 (0.37~0.96)	0.034*	0.67 (0.42~1.04)	0.075
GDR score						
Continuous	0.96 (0.88~1.05)	0.366	0.97 (0.89~1.05)	0.446	0.99 (0.91~1.07)	0.752
Categories						
≤-3	1.00 (reference)	-	1.00 (reference)	-	1.00 (reference)	-
-2~0	0.79 (0.51~1.21)	0.274	0.77 (0.50~1.19)	0.249	0.84 (0.55~1.26)	0.402
≥1	0.66 (0.37~1.19)	0.168	0.75 (0.42~1.35)	0.335	1.03 (0.60~1.77)	0.916
Dietary Diversity Score						
Continuous	0.98 (0.96~1.00)	0.057	0.98 (0.96~1.00)	0.032*	0.98 (0.96~1.00)	0.034*
Categories						
≤10	1.00 (reference)	-	1.00 (reference)	-	1.00 (reference)	-
11~20	0.76 (0.47~1.24)	0.276	0.62 (0.37~1.01)	0.055	0.83 (0.52~1.31)	0.418
≥21	0.66 (0.41~1.04)	0.071	0.71 (0.45~1.11)	0.131	0.67 (0.43~1.03)	0.070

Supplementary Table S12. Associations of diet quality indicators with AS, KOA, and AS+KOA relative to the Reference group among men.

Diet quality indicators	AS		KOA		AS+KOA	
	OR (95% CI)	P value	OR (95% CI)	P value	OR (95% CI)	P value
NCD-Protect score						
Continuous	0.94 (0.88~1.00)	0.035*	0.92 (0.86~0.98)	0.013*	0.95 (0.90~1.01)	0.088
Categories						
≤3	1.00 (reference)	-	1.00 (reference)	-	1.00 (reference)	-
4~6	0.89 (0.54~1.47)	0.650	0.87 (0.54~1.42)	0.573	0.85 (0.54~1.35)	0.494
≥7	0.64 (0.39~1.04)	0.070	0.72 (0.46~1.12)	0.145	0.77 (0.50~1.18)	0.226
NCD-Risk score						
Continuous	0.96 (0.90~1.02)	0.168	0.94 (0.88~1.00)	0.042*	0.95 (0.89~1.00)	0.052
Categories						
≤4	1.00 (reference)	-	1.00 (reference)	-	1.00 (reference)	-
5~8	0.85 (0.52~1.39)	0.518	0.82 (0.51~1.32)	0.423	0.76 (0.49~1.19)	0.235
9	0.53 (0.31~0.91)	0.021*	0.60 (0.36~0.99)	0.045*	0.61 (0.38~0.97)	0.038*
GDR score						
Continuous	0.95 (0.87~1.04)	0.311	0.97 (0.88~1.07)	0.550	1.02 (0.94~1.11)	0.630
Categories						
≤-3	1.00 (reference)	-	1.00 (reference)	-	1.00 (reference)	-
-2~0	0.60 (0.37~0.97)	0.038*	0.72 (0.44~1.15)	0.168	0.81 (0.51~1.25)	0.342
≥1	0.66 (0.34~1.27)	0.217	0.66 (0.35~1.26)	0.210	1.19 (0.67~2.15)	0.554
Dietary Diversity Score						
Continuous	0.98 (0.96~1.00)	0.082	0.97 (0.95~1.00)	0.018*	0.98 (0.96~1.00)	0.044*
Categories						
≤10	1.00 (reference)	-	1.00 (reference)	-	1.00 (reference)	-
11~20	0.76 (0.44~1.31)	0.320	0.69 (0.40~1.17)	0.168	0.79 (0.48~1.30)	0.356
≥21	0.58 (0.34~0.95)	0.032*	0.70 (0.43~1.12)	0.141	0.67 (0.42~1.06)	0.089

Supplementary Table S13. Associations of diet quality indicators with AS, KOA, and AS+KOA relative to the Reference group among women.

Diet quality indicators	AS		KOA		AS+KOA	
	**OR (95% CI)**	**P value**	**OR (95% CI)**	**P value**	**OR (95% CI)**	**P value**
NCD-Protect score						
Continuous	0.96 (0.80~1.13)	0.601	1.01 (0.87~1.17)	0.885	0.91 (0.77~1.08)	0.299
Categories						
≤3	1.00 (reference)	-	1.00 (reference)	-	1.00 (reference)	-
4~6	0.97 (0.32~2.92)	0.962	1.09 (0.31~3.93)	0.888	0.71 (0.21~2.37)	0.575
≥7	1.12 (0.37~3.35)	0.834	0.51 (0.13~1.86)	0.312	0.42 (0.12~1.43)	0.167
NCD-Risk score						
Continuous	0.98 (0.84~1.15)	0.781	1.02 (0.89~1.17)	0.759	1.03 (0.88~1.22)	0.676
Categories						
≤4	1.00 (reference)	-	1.00 (reference)	-	1.00 (reference)	-
5~8	0.78 (0.27~2.17)	0.645	0.74 (0.21~2.51)	0.621	0.81 (0.21~3.00)	0.748
9	0.97 (0.29~3.18)	0.957	0.71 (0.17~2.84)	0.628	1.13 (0.29~4.42)	0.857
GDR score						
Continuous	0.96 (0.74~1.23)	0.724	0.97 (0.78~1.21)	0.817	0.77 (0.59~0.98)	0.037*
Categories						
≤-3	1.00 (reference)	-	1.00 (reference)	-	1.00 (reference)	-
-2~0	2.89 (1.06~8.14)	0.037*	1.10 (0.33~3.78)	0.879	1.40 (0.46~4.39)	0.557
≥1	0.72 (0.20~2.61)	0.616	0.86 (0.19~3.73)	0.843	0.19 (0.02~1.11)	0.066
Dietary Diversity Score						
Continuous	0.98 (0.93~1.04)	0.522	1.00 (0.95~1.05)	0.999	0.98 (0.92~1.03)	0.410
Categories						
≤10	1.00 (reference)	-	1.00 (reference)	-	1.00 (reference)	-
11~20	0.83 (0.25~2.52)	0.741	0.36 (0.08~1.52)	0.167	0.92 (0.23~3.51)	0.904
≥21	1.17 (0.36~3.65)	0.791	0.82 (0.23~2.85)	0.755	0.57 (0.14~2.18)	0.417

Supplementary Table S14. Sensitivity analysis with additional adjustment for hyperlipidemia and elevated LDL-C using Firth penalized logistic regression.

Diet quality indicators	Total (n=1538)		Men (n=1340)		Women (n=198)	
	OR (95% CI)	P value	OR (95% CI)	P value	OR (95% CI)	P value
NCD-Protect score						
Continuous	1.00 (0.96~1.03)	0.859	1.00 (0.97~1.04)	0.842	0.90 (0.79~1.03)	0.121
Categories						
≤3	1.00 (reference)	-	1.00 (reference)	-	1.00 (reference)	-
4~6	1.06 (0.81~1.38)	0.678	1.06 (0.80~1.40)	0.698	0.66 (0.26~1.61)	0.363
≥7	0.95 (0.74~1.23)	0.694	1.01 (0.78~1.32)	0.918	0.41 (0.15~1.05)	0.063
NCD-Risk score						
Continuous	1.00 (0.97~1.03)	0.943	0.99 (0.96~1.03)	0.693	1.04 (0.92~1.19)	0.570
Categories						
≤4	1.00 (reference)	-	1.00 (reference)	-	1.00 (reference)	-
5~8	0.94 (0.72~1.21)	0.619	0.91 (0.70~1.19)	0.491	1.01 (0.38~2.82)	0.977
9	1.02 (0.77~1.35)	0.892	0.97 (0.72~1.30)	0.826	1.41 (0.51~4.12)	0.511
GDR score						
Continuous	1.00 (0.95~1.05)	0.878	1.02 (0.97~1.08)	0.373	0.77 (0.64~0.92)	0.003*
Categories						
≤-3	1.00 (reference)	-	1.00 (reference)	-	1.00 (reference)	-
-2~0	0.96 (0.75~1.25)	0.777	1.04 (0.80~1.37)	0.755	0.63 (0.28~1.42)	0.260
≥1	1.16 (0.83~1.62)	0.393	1.34 (0.94~1.92)	0.101	0.18 (0.03~0.77)	0.019*
No. of DQQ food groups						
Continuous	1.00 (0.99~1.01)	0.649	1.00 (0.99~1.01)	0.754	0.98 (0.94~1.02)	0.349
Categories						
≤10	1.00 (reference)	-	1.00 (reference)	-	1.00 (reference)	-
11~20	1.25 (0.94~1.66)	0.131	1.19 (0.88~1.61)	0.250	1.08 (0.41~2.94)	0.875
≥21	0.95 (0.73~1.24)	0.690	0.97 (0.74~1.28)	0.849	0.56 (0.20~1.58)	0.267

Supplementary Figure S1. Distribution of DQQ-derived diet quality scores among 1,538 participants. Bars show the number of participants at each integer score. Dotted vertical lines indicate the category boundaries used in the categorical analyses.

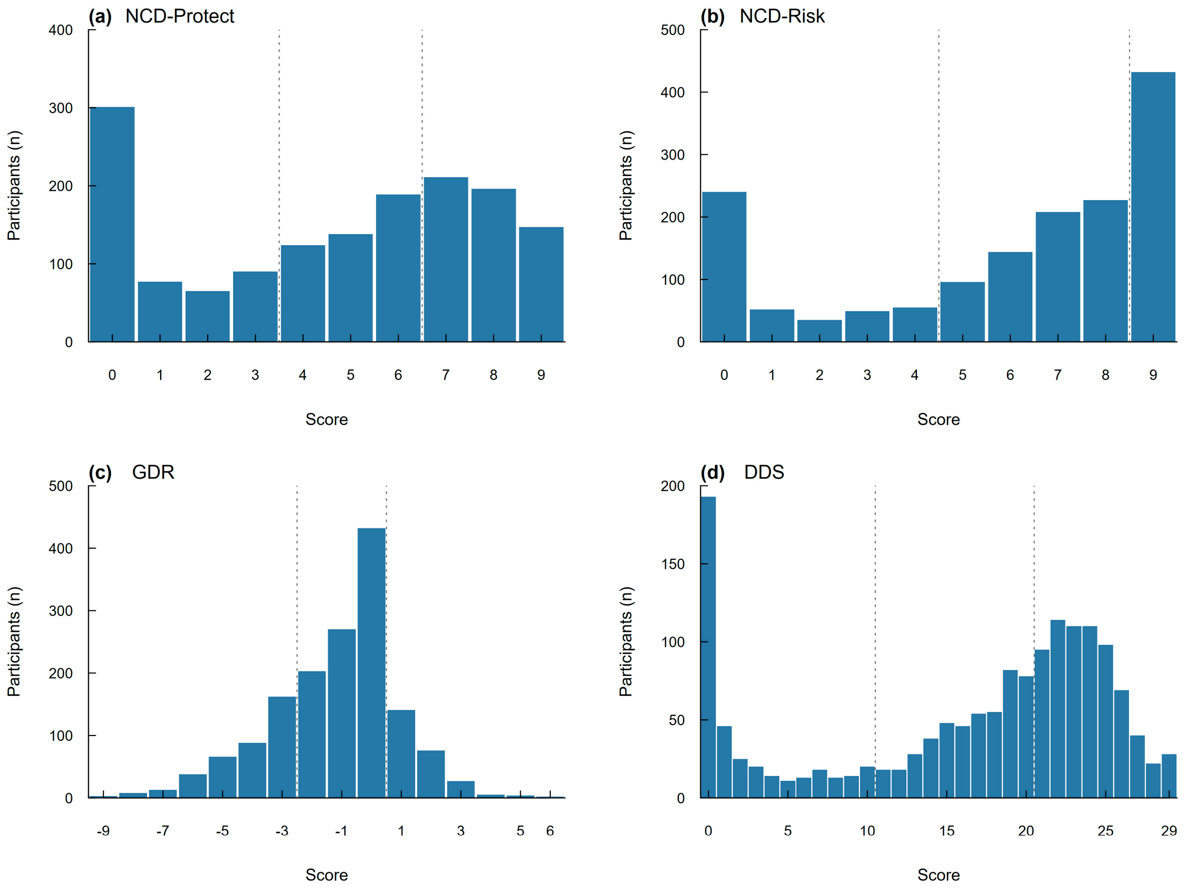

Supplement: Supplementary file 1 [file nutrients-18-03352-s001.zip › nutrients-4484344-supplementary.pdf]
